# Pre-trained deep learning models for brain MRI image classification

**DOI:** 10.3389/fnhum.2023.1150120

**Published:** 2023-04-20

**Authors:** Srigiri Krishnapriya, Yepuganti Karuna

**Affiliations:** School of Electronics Engineering, Vellore Institute of Technology, Vellore, India

**Keywords:** convolutional neural networks, transfer learning, VGG-19, VGG-16, inception V3, ResNet50

## Abstract

Brain tumors are serious conditions caused by uncontrolled and abnormal cell division. Tumors can have devastating implications if not accurately and promptly detected. Magnetic resonance imaging (MRI) is one of the methods frequently used to detect brain tumors owing to its excellent resolution. In the past few decades, substantial research has been conducted in the field of classifying brain images, ranging from traditional methods to deep-learning techniques such as convolutional neural networks (CNN). To accomplish classification, machine-learning methods require manually created features. In contrast, CNN achieves classification by extracting visual features from unprocessed images. The size of the training dataset had a significant impact on the features that CNN extracts. The CNN tends to overfit when its size is small. Deep CNNs (DCNN) with transfer learning have therefore been developed. The aim of this work was to investigate the brain MR image categorization potential of pre-trained DCNN VGG-19, VGG-16, ResNet50, and Inception V3 models using data augmentation and transfer learning techniques. Validation of the test set utilizing accuracy, recall, Precision, and F1 score showed that the pre-trained VGG-19 model with transfer learning exhibited the best performance. In addition, these methods offer an end-to-end classification of raw images without the need for manual attribute extraction.

## 1. Introduction

The brain is one of the most complicated organs with billions of cells. A brain tumor is an abnormal collection of cells brought on by unrestricted cell division in the brain. This peculiar group of cells might damage healthy cells and reduce brain function if safeguards are not taken in a timely manner. Like all other forms of cancer, brain tumors are a major threat to human life and may be fatal if they are not detected and treated in a timely manner. In 2019, 24,000 new cases of brain cancer were discovered in the US (Cancer.Net, 2020). The effective management of brain tumors depends on an early and precise diagnosis. As computer-assisted diagnosis (CAD) benefits neuro-oncologists in vast directions, the detection of brain tumors using CAD is a keen area for research (Kumar et al., 2017).

Deep and machine-learning-based diagnostic systems are examples of CAD. Magnetic resonance (MR) imaging is the foundation of CAD systems. Magnetic resonance imaging (MRI), is one of the many medical imaging methods frequently used to image abnormal brain tissues. Among the other methods, MRI is the most widely used and safest imaging technique. Compared with CT images, MRI shows more contrast in the soft tissues of the brain. High-resolution brain data are available from MRI. Abnormalities in the brain can be quickly detected using MRI.

Advancements in Medical imaging allow for an in-depth examination of human organs using a large number of images. Radiologists typically scan MR images to detect irregularities in the brain. Despite improvements in hardware and medical imaging procedures, it is difficult to manually evaluate the vast volumes of data generated by MRI. This has led to the emergence of computer-aided semi-automatic or fully-automatic image analysis as a significant research topic (Bauer et al., 2013). High levels of precision and clarity are required because this issue has a direct impact on human health. At this stage, computer-aided diagnostics assist in obtaining precise results quickly.

The goal of brain tumor segmentation is to locate and map tumors as well as any effective tumor tissue and edema. This is accomplished by contrasting uneven tissues with normal tissues. Brain tumor segmentation using MR images is crucial for developing an advanced diagnosis and treatment strategy. Most current generative or discriminative model-based techniques for segmenting brain tumors are automated or semi-automated (Menze et al., 2014). Understanding probabilistic image atlases of both healthy and malignant tissues is necessary for generative models. The functionality of the method relies on the image qualities and the classification processes utilized, and exemplary methods categorize MR images as either tumors or normal tissues based on their features. Machine learning methods are utilized in discriminative models to handle handcrafted features. A collection of algorithms called “machine learning” enables computers to anticipate outcomes from large amounts of data. However, these strategies require specialized knowledge to extract features that permit classification (Akkus et al., 2017).

Artificial intelligence (AI) in healthcare uses complex algorithms and software to estimate human cognition in the analysis of complicated medical data. AI technology is distinguished from traditional technologies in health care by its ability to gain information, process it, and give a well-defined output to the end-user. There has been an increase in research on AI in various specialties in medicine such as Radiology, Imaging, Telehealth, Electronic Health Records, and Industry. Transfer learning is a research problem in machine learning that focuses on storing the knowledge gained while solving one problem and applying it to a different but related problem. One of the fundamental requirements of transfer learning is the presence of models that perform well on source tasks. Fortunately, the deep learning world believes in sharing. Many state-of-the-art deep-learning architectures have been openly shared by their respective teams.

Deep learning is specific categories of algorithms that have been utilized to successfully reap the benefits of transfer learning. A convolutional neural network (CNN or ConvNet) is one of the most popular algorithms for deep learning, a type of machine learning in which a model learns to perform classification tasks directly from images, videos, text, or sound. CNNs are particularly useful for identifying patterns in images to recognize objects, faces, and scenes. They learn directly from image data using patterns to classify images, and eliminate the need for manual feature extraction. Applications that call for object recognition and computer vision, such as self-driving vehicles and face recognition applications, rely heavily on CNNs. Depending on the application, a CNN can be built from scratch or a pre-trained model can be used with the dataset. For image recognition tasks, the use of pretrained models was significant. For one, they are easier to use as they give you the architecture for “free.” Additionally, they typically have better results and require less training.

In the literature, many MR image categorization methods have been used for brain abnormalities. These investigations often employ preprocessing, feature extraction, and classification stages to distinguish between normal and abnormal images. Many supervised machine learning techniques have been used in these studies, including Independent Component Analysis (ICA), Support Vector Machines (SVM), Random Forest Classifiers (RFC), and effective Gaussian mixture models (GMM) (Domingues et al., 2018). Modern reviews employ cutting-edge machine-learning methods. The Discrete Wavelet Transform (DWT) was applied by Nayak et al. for feature extraction, feature reduction, and the AdaBoost algorithm with RFC (Nayak et al., 2016). The same researchers improved the results in a different study by creating a model that makes use of the SVM with AdaBoost (Nayak et al., 2018).

To train the neural network aimed at the categorization of brain cancers, Mohsen et al. applied the characteristics obtained by the Principal Component Analysis (PCA) and DWT approach to segmented MR brain images (Mohsen et al., 2018). Zhang et al. created a brain MRI classifier by fusing Particle Swarm Optimization (PSO) and SVM. In a different study, the same researchers substituted the wavelet entropy approach for the SVM (Zhang et al., 2015). Wang et al. applied feed-forward neural networks, PSO, and artificial bee colonies (Wang et al., 2015). Probabilistic neural networks (PNN) and the wavelet entropy approach were employed by Saritha et al. (2013).

However, traditional machine learning algorithms are not adept at making generalizations. Deep learning, a machine learning method that has gained a lot of attention recently, overcomes the drawbacks of traditional machine learning algorithms and, owing to its capacity for self-learning, enables autonomous feature detection in MR images. Deep learning requires less feature engineering because features are inevitably extracted by different processing layers (Ahmad and Choudhury, 2022). A variety of issues can be solved using deep-learning approaches.

With the advent of the idea of “deep learning”, contemporary studies on CAD indicate enhanced performance, and the effectiveness of deep learning is accelerating in the research field (Cao et al., 2018). Deep learning archetypes were employed in a variety of biomedical utilizations, including the analysis of pulmonary nodules (Cheng et al., 2016), skin cancer (Zuo et al., 2017), breast cancer (Yousefi et al., 2018), lung cancer (Gu et al., 2018), and histopathological diagnostics (Litjens et al., 2016). Owing to differences in brain shape, imaging methods, and equipment, automated detection of brain disorders continues to be a challenge despite diligent studies in medical image processing.

Convolutional neural networks (CNN) work well with deep-learning-based techniques for the segmentation of brain tumors. CNN or Deep CNN (DCNN) can automatically extract features from brain MR images by parameter tuning of the convolutional (“conv”) and the pooling (“pool”) layers, and the model had an 81 percent classification accuracy (Charron et al., 2018). The CNN models have been successfully applied to several biomedical applications. Agn et al. suggested a deep learning-based approach to modify radiotherapy planning parameters for brain tumors to reduce the risk to healthy tissues (Agn et al., 2019). To classify brain cancers, Afsharet al. changed the CNN design with a capsule neural network, but the performance gain was insufficient (Afshar et al., 2018).

The classification capability of a CNN is highly reliant on the size of the data used for training. If the size of the dataset is small, the CNN starts overfitting. In this scenario, the concept of transfer learning has emerged, and it has proven to be effective for classification tasks. Transfer learning is the process of applying the understanding of one pre-trained neural network to another, however, it is a new model. The diagnosis of medical issues using computers offers great promise for transfer learning. To identify kidney cancer, a pre-trained Inception V3 model was employed (Zhou et al., 2019). The VGG-16 and AlexNet models were employed to diagnose breast cancer, and then SVM was used to classify tumors (Deniz et al., 2018).

A transfer learning model for the classification of pancreatic and lung tumors was proposed by Hussein et al. (2019). Studies on neuro-oncology have also focused on transfer learning. According to a study conducted by researchers, transfer learning is superior to conventional machine learning for the classification of brain tumors (Kaur and Gandhi, 2020). AlexNet and GoogLeNet have been used to grade tumors on brain MR images (Yang et al., 2018). Using MR images, a pre-trained VGG-16 deep learning network was employed to identify Alzheimer's illness (Jain et al., 2019). Using MR images, a trained GoogLeNet was used to distinguish between the three different types of cancer (Deepak and Ameer, 2019). For a comparable objective, a study was conducted using the AlexNet, GoogLeNet, and VGG models (Rehman et al., 2020). A revision was made to represent the efficiency of transfer learning with constrained time and a few epochs in classifying brain tumors (Chelghoum et al., 2020).

In transfer learning, the weights of the “conv” layer from pre-trained models are used and only the last layers are trained with data from the newer classes. In the proposed work, the capability of four well-known distinct pre-trained CNN models with transfer learning was investigated for the automated classification of 305 brain MR images into tumorous and non-tumorous categories. Accuracy, recall, sensitivity, and F1-score measurements were used to assess the VGG-19, VGG-16, ResNet50, and Inception V3 models. The goal was to attain high accuracy values in small epochs with a relatively small dataset. The findings of this study were compared with those of recent studies. The success rate of the VGG-19 model was higher than that reported in comparable studies in the literature. This work provides an alternate and effective method for the detection of tumors in MRI brain images.

The key offerings of the proposed article are summarized as follows:

- The performance of four well-known unique pre-trained CNN models with transfer learning was examined for the automatic categorization of 305 brain MR images into tumorous and non-tumorous categories.
- The performance of the models was measured using a variety of performance parameters, namely accuracy, recall, sensitivity, and F1-score.
- High accuracy values were achieved with fewer epochs and a smaller dataset.

This paper comprises four primary sections. Materials and methods section describes the materials and methods used. The experimental results are presented in results section. The discussion is presented in Discussions section. Finally, Conclusion section concludes the work.

## 2. Materials and methods

The majority of earlier investigations relied on manually extracting tumor behaviors before classifying them. However, they cannot become totally automated because of this method. If the amount of data is relatively small, only a few studies have demonstrated the production of solutions.

This article used the transfer learning process in deep convolutional neural networks to propose an automated classification approach for brain MRI data. A total of 305 brain MRI images were included in the dataset after augmentation. The images were preprocessed prior to the deep neural network learning processes. The fundamental steps of the proposed method are as follows:

1. ***Pre-processing***: With the use of the Open CV library, raw MRI images have been cropped, and the boundaries of the brain tissue were identified.
2. ***Deep learning model implementations***: VGG-19, VGG-16, ResNet50, and InceptionV3 were the four separate pre-trained models that were used.
3. ***Data augmentation***: Data augmentation techniques were used during training to improve learning outcomes and avoid overfitting.
4. Several criteria, including accuracy, precision, recall, and F1 score, were used to assess the performance of the models.

**Figure 1:**
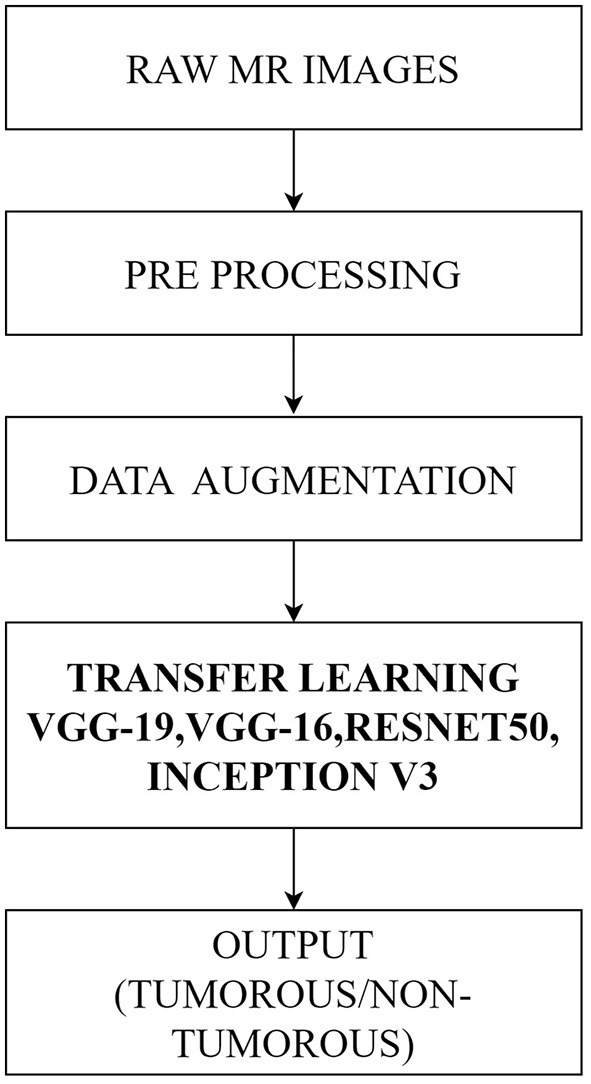
Flowchart of the proposed methodology using pre-trained DCCN models.

Figure 1 illustrates the workflow of the proposed method.

### 2.1. Dataset

**Figure 2:**
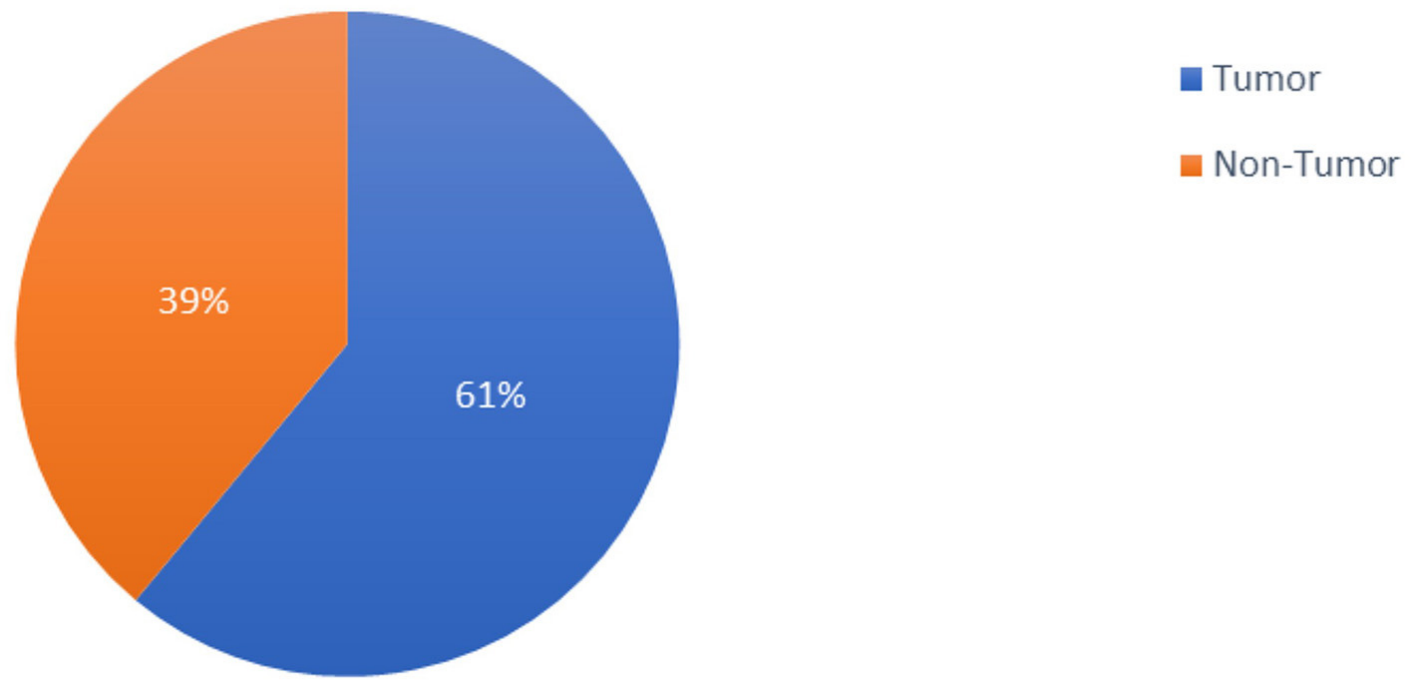
Pie chart showing the distribution of tumorous and non-tumorous images in the dataset.

The 253 brain MRI scans that Chakrabarty gathered and made publicly available constituted the dataset used in the study (Chakrabarty, 2019). The 253 brain MRI scans that comprised the study's dataset were total in number. Among these images, 98 had no tumors, and 155 had tumors. Figure 2 provides a graph illustrating the dataset distributions of the tumor and non-tumor images.

The distribution of the image classes within the dataset was unbalanced. The classifier is more likely to select the dominant class as a result of the dominance of the class in the dataset. The number of cases of the prevailing class should be decreased or the data class with fewer instances must be increased to solve the instability issue. Plummeting the amount of data from the dominant class reduced the generalizability of the classification because the dataset utilized in this investigation was rather small. Thus, data augmentation was applied to the classes with an insufficient number of samples.

**Figure 3:**
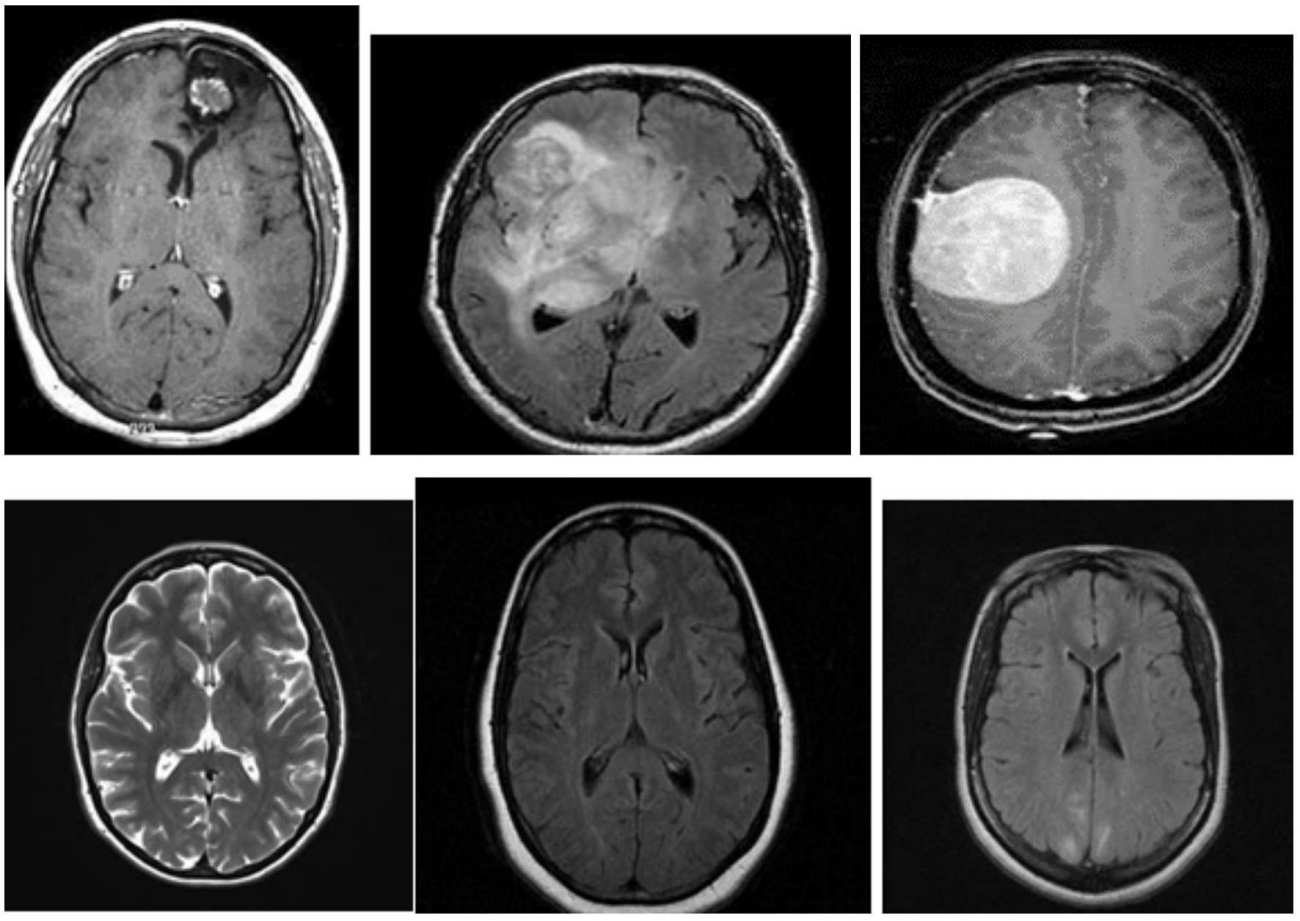
MR images with and without tumor from the dataset.

Figure 3 shows examples of MR images of the dataset with and without malignancies. The black frames surrounding the images in the dataset have varying sizes. Pre-processing was performed to normalize the images before training.

### 2.2. Image pre-processing and data augmentation

**Figure 4:**
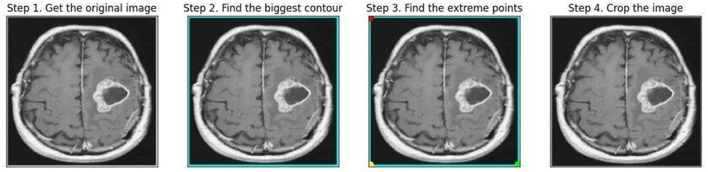
Steps involved in preprocessing.

The endpoints at four distinct ends, left, right, top, and bottom was recognized as the borderlines of the brain tissue all over the frame surrounding the image during preprocessing using the OpenCV package with Python, as displayed in Figure 4.

Polar points and borders were used to crop the images. The borders of the brain tissue were established based on the raw image, and the exterior portion was eliminated. This facilitated data processing. The images had different shapes (e.g., 630X630, 225X225). After cropping, the MR images with varying length and width values were resized to 224 × 224 pixels. Overfitting results from working with a dataset that is unbalanced and contains a minimal amount of data. The model is prone to learning the dominant class and is therefore unable to be applied to different contexts. This issue is avoided by using a data augmentation technique that also diversifies the data. By performing operations on the current dataset at different rates, such as zooming, reflecting, and rotating, the data augmentation technique makes it possible to generate new data (Khan et al., 2021). Depending on the dataset and procedure, empirical data are used to determine the transformation procedures and rates employed for the original data. In this analysis, the present dataset was enhanced using 15% rotation, 5% horizontal and vertical axis shifting, 1/255 rescaling, and horizontal and vertical axis mirroring. This study's rotation, shifting, and scaling ratios were also acquired empirically in a model that maximizes its work improvement. To balance the collection, data augmentation was used to recreate non-tumor images using a smaller number of samples. The number of non-tumor images increased by 50%, whereas the number of images with tumors remained constant. The number of images in each class as a result of data augmentation is shown in Table 1.

**Table 1:** Images count after data augmentation.

**Class**	**Count**
Tumor images	155
Non-tumor images	150
Total	305

### 2.3. Deep learning, CNN, and transfer learning

Deep learning is a technique that separates a feature hierarchy from input data using multiple-layer neural networks. It is a well-liked technique, as opposed to manual feature extraction in standard machine learning, which automatically extracts features from images. Various deep-learning algorithms have been employed to achieve various objectives.

**Figure 5:**
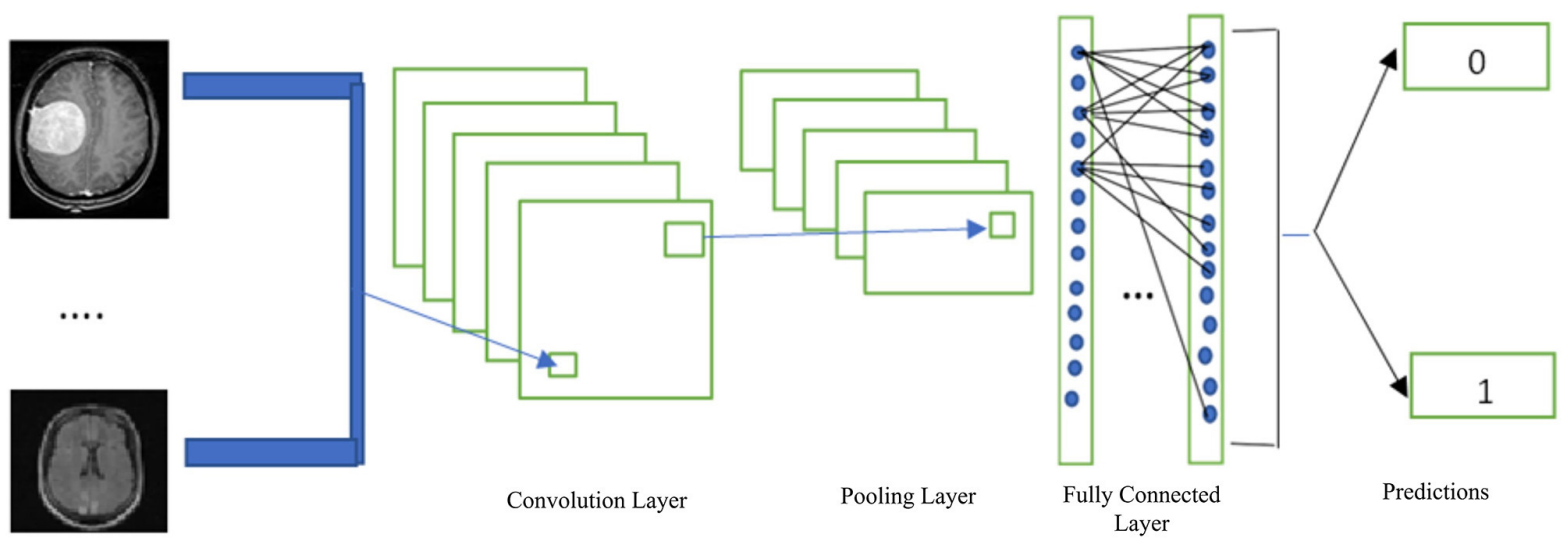
Basic CNN architecture (Hinton, 2012).

A CNN is a deep learning method that is frequently employed in image classification and segmentation. In addition, CNN is frequently utilized in the analysis of medical images to provide improved outcomes (Belaid and Loudini, 2020). CNN offers an automated feature definition and extraction from images. The convolution, pooling, activation, and classification layers are common components of CNN. The CNN receives images that have been categorized using specified tags, which are then used to enhance the trainable parameters in the system to improve classification accuracy. The input pixels are subjected to kernel application in the convolution layer, which reveals the features. By using the average or largest of the data from the earlier levels, the pooling layer minimizes the data. Transfer to the prediction phase is made possible by the fully connected layer. Figure 5 depicts the general layout of the CNN architecture.

In each convolutional layer, a Rectified Linear Unit (ReLU) activation function was used. The input-weighted sum is transformed into the output of the node through an activation function (Pedada et al., 2023). The rectifier linear unit function was utilized in the hidden layers of the CNN. ReLU is denoted mathematically by


(1) $$\begin{array}{c} f\left(x\right)=\max\left(0,x\right) \end{array}$$

Here, ‘x' represents the input. The negative input is converted to zero when x is negative or equal to zero. Inputs larger than zero result in an output of 1. Therefore, ReLUs' derivative will be


(2) $$\begin{array}{l} f^{\prime }\left(x\right)=1,\;for\;x\geq 0\\ 0,for\;x\;<0 \end{array}$$

As a result, the neuron is a dead neuron in the ReLU function and is not activated if the input is 0.

#### 2.3.1. Loss function

In machine learning, the error between the actual label values and algorithmically predicted values is calculated using the loss function. Subsequently, using any optimization technique, the error was reduced to a minimum value. The cross-entropy loss function was used in this work (Mannor et al., 2005). We utilized binary cross entropy because we were binary in classifying our MRI images. The cross-entropy error rate in binary calculations ranged from 0 to 1. In mathematics, it is denoted as


(3) $$\begin{array}{c} J\left(x\right)=xlogP\left(x\right)+\left(1-x\right)\log\left(1-P\left(x\right)\right) \end{array}$$

In this case, *P(x)* is the predicted label, and *x* is the actual label. Because *x* is multiplied by log, the first term will be zero when the actual labels, *x*, are equal to 0. Similarly, when *x* = 1, the second component is equal to zero. *J(x)* will be 0 if *x* = *P(x)*.

#### 2.3.2. Optimization

To reduce the loss in deep neural networks, we applied several optimization techniques by adjusting variables such as weights and learning rates. We employed Adaptive Moment Estimation (Adam) optimizer in our research, suggested by Kingma and Ba (2014). The Adam optimizer combines the momentum-based stochastic gradient descent with RMSprop.

H. Robbins presented the Stochastic Gradient Descent technique (Robbins and Munro, 1951). In the stochastic gradient descent, we calculate the derivatives of the weights (*dw*) and bias (*dB*) for each epoch. Then multiply by the learning rate.


(4) $$\begin{array}{l} w=w-\eta \times dw\\ B=B-\eta \times dB \end{array}$$

When we calculated *dw* and *dB* for the current batch, we obtained moving mean between 0 and 1. Stochastic gradient descent with momentum U is the moving mean of our gradients.


(5) $$\begin{array}{l} U_{dw}=\Gamma \times U_{dw}+(1-\;\Gamma )\times dw\\ U_{dB}=\Gamma \times U_{dB}+\left(1-\;\Gamma \right)\times dB \end{array}$$

Similarly, Hinton proposed an adaptive learning rate algorithm known as the Root Mean Squared Prop. We use the exponential moving mean square of the gradients in RMSProp. RMSProp is represented mathematically as,


(6) $$\begin{array}{l} R_{dw}=\Gamma \times R_{dw}+(1-\;\Gamma )\times dw^{2}\\ R_{dB}=\Gamma \times R_{dB}+\left(1-\;\Gamma \right)\times \;dB^{2} \end{array}$$

A hyperparameter called gama Γ adjusts the exponentially weighted means. To use the Adam optimization technique, we combined the characteristics of the weighted mean and the weighted mean of the squares of the previous gradients. Consequently, Adam Optimizer's revised weights and bias will be


(7) $$\begin{array}{l} w=w-\eta \times (U_{dw}/\sqrt{R_{dw}+\;R})\\ B=B-\eta \times \;(U_{dB}/\sqrt{R_{dB}+R}) \end{array}$$

Epsilon R (Epsilon = 10–8) eliminates zero division and η represents the learning rate.

Transfer learning involves transferring weights from a network that has been extensively trained with data to another model designed to address a related issue (Podder et al., 2021). If there is insufficient training data for the current problem, this strategy becomes crucial. The network may experience an overfitting issue with a limited amount of data, making generalization impossible. The parameters of the transferred network guarantee the accurate categorization of a small volume of data if the dataset used to train the pretrained model is sufficiently large. Only the classifiers in the last section of the new model were trained, and the estimated weights from the pretrained model were transferred to it.

Transfer learning is advantageous when few images are available, according to studies on medical image analysis (Deepak and Ameer, 2020). In this study, pretrained VGG-19, VGG-16, ResNet50, and Inception V3 transfer learning models were used to execute a brain MRI classification task, and the performance of the models were compared. Models were trained by 25 epochs for each fold, and a high level of classification performance was achieved.

#### 2.3.3. VGG-19 model

VGG-19 is a variant of the VGG model that consists of 19 layers (16 convolution layers, three fully connected layers, five MaxPool layers, and one SoftMax layer). Other variants of VGG like VGG-11, VGG-16, and others.

**Figure 6:**
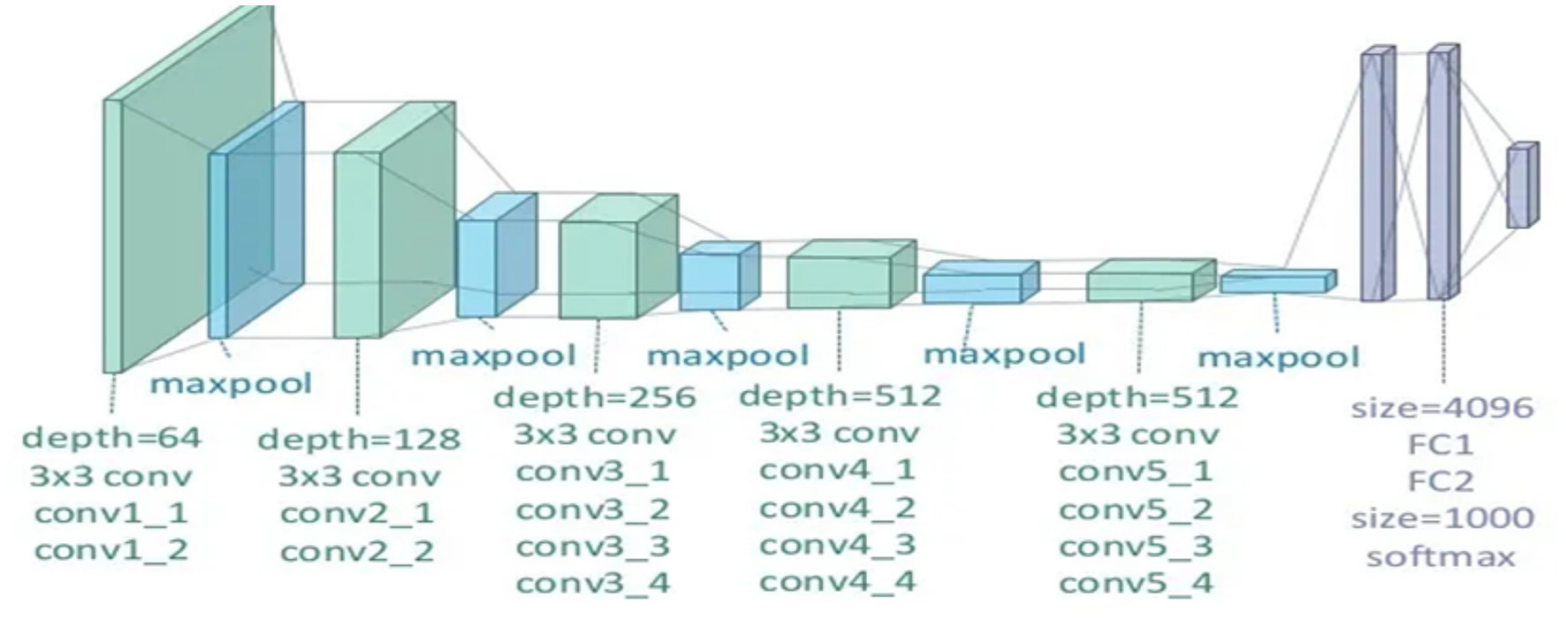
Illustration of the network architecture of VGG-19 model (Abuared et al., 2020).

Generally, the pre-trained VGG-19 model involves 19 layers, 16 are convolutional, and three are fully connected layers (Latha et al., 2021). In VGG-19, the first and second convolution layers of the group of two layers are followed by max-pooling layers, and the next eight convolution layers are a group of four layers followed by max-pooling with the same filter size, that is, 3 × 3; the last three are dense layers, containing 4,096 images and 1,000 features followed by the SoftMax function, as shown in Figure 6.

#### 2.3.4. VGG-16 model

**Figure 7:**
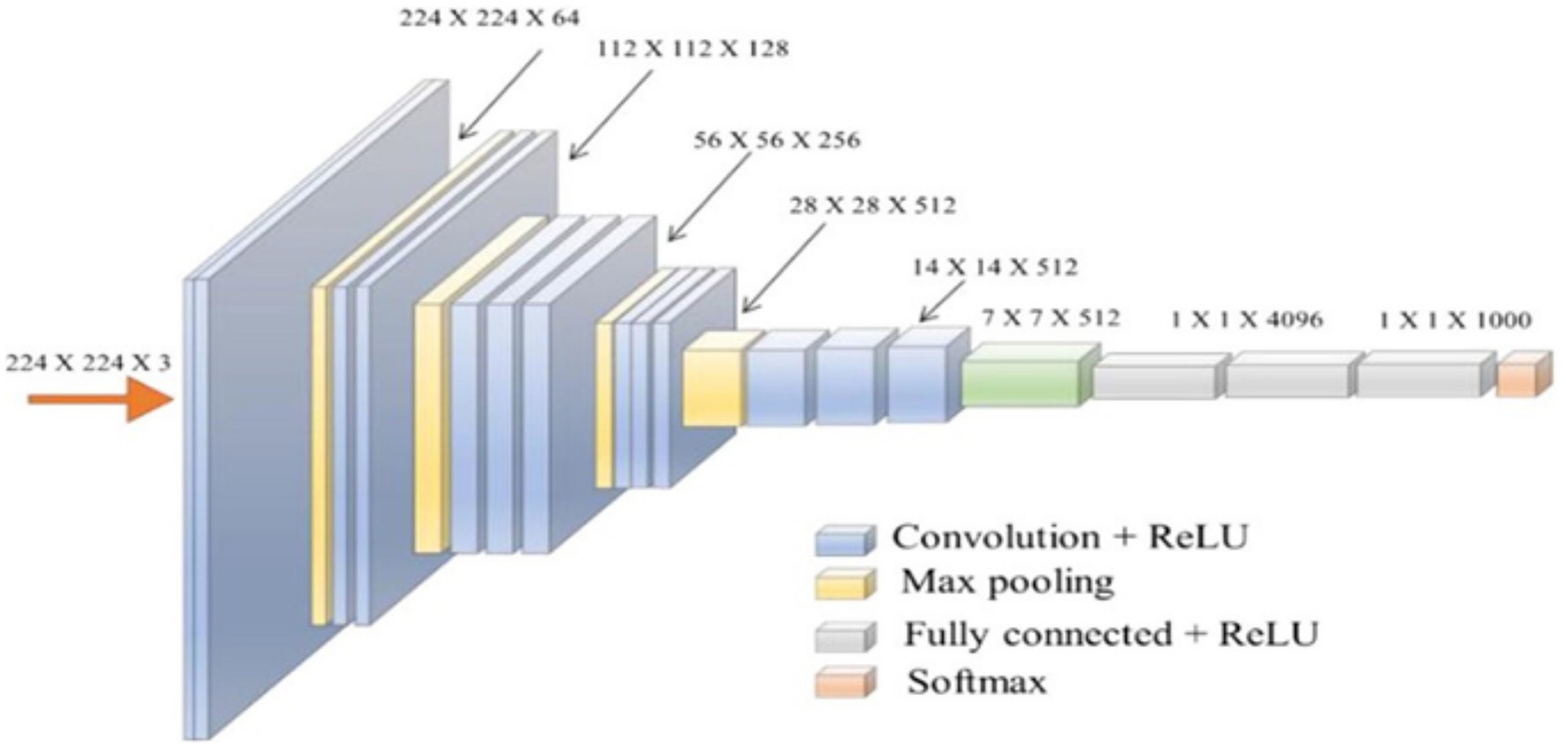
The standard VGG-16 network architecture (Simonyan and Zisserman, 2014).

Simonyan and Zisserman's VGG network architecture is a CNN model first presented in 2014 (Simonyan and Zisserman, 2020). The unique VGG type, known as VGG-16 had 16 weighted layers. Convolution, maximum pooling, activation, and fully linked layers are the layers. Figure 7 depicts the general framework of the VGG-16 model.

The design has 21 layers in total; 13 convolutional layers, five pooling layers, and three dense layers. There were only16 weighted layers among them. There are 64 filters in the first convolution layer, 128 filters in the second layer, 256 filters in the third layer, and 512 filters in each of the fourth and fifth levels. The ImageNet dataset, which contains more than 14 million images and 1,000 classifications, was used to train the VGG-16 network (Ghosh et al., 2021), which achieved an accuracy rate of 92.7%.

#### 2.3.5. ResNet50 model

**Figure 8:**
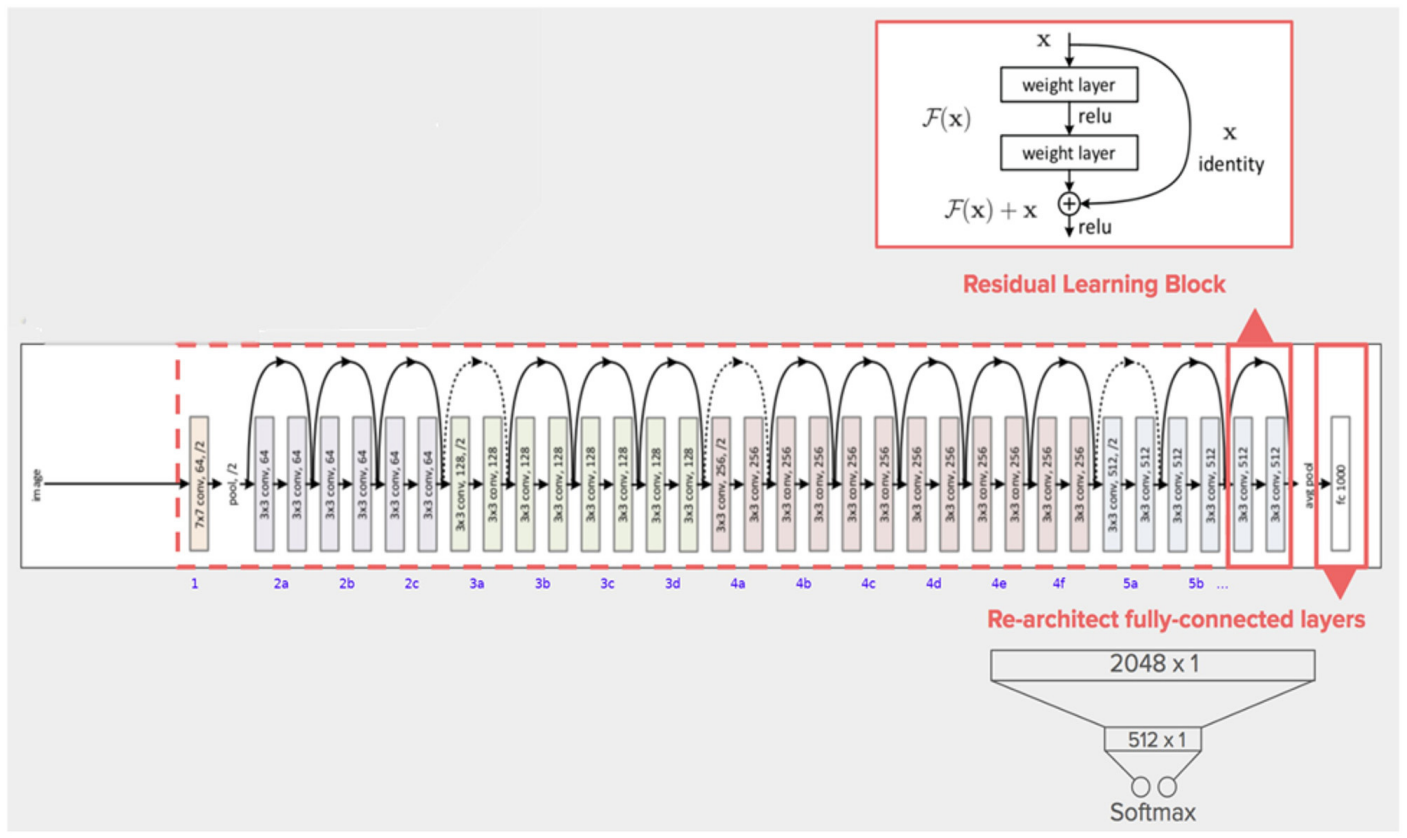
The architecture of ResNet50 model (Khan et al., 2021).

He et al. introduced ResNet in 2015, and with a 3.57% error rate, their model won the ImageNet competition (He et al., 2016). ResNet's structure is based on microarchitecture modules, in contrast to conventional sequential network topologies. Theoretically, as a model's number of layers increases, its success should follow. However, as the number of factors increases, training and optimization become increasingly challenging. In deep neural networks, neurons with low activation are useless during training; in these cases, residues develop in the network. Blocks that feed subsequent layers with residual values were added to construct ResNet as shown in Figure 8.

In a typical CNN, a non-linear G (x) function is used to represent a model that proceeds sequentially from the input to the output. In ResNet, “x” is the input value, which is summed arithmetically to the function F(x) by making a crosscut from the input to the output. The idea behind this network is to enable it to fit the residual mapping instead of having layers learn the underlying mapping.

F (x) + x is then transmitted through the ReLU. The input is appended after the completion of the second layer, with the intention of more forcefully communicating the values in the previous levels to the following layers. The ResNet50 is a unique ResNet model that has fifty weighted layers (Kumar et al., 2022).

#### 2.3.6. Inception V3 model

**Figure 9:**
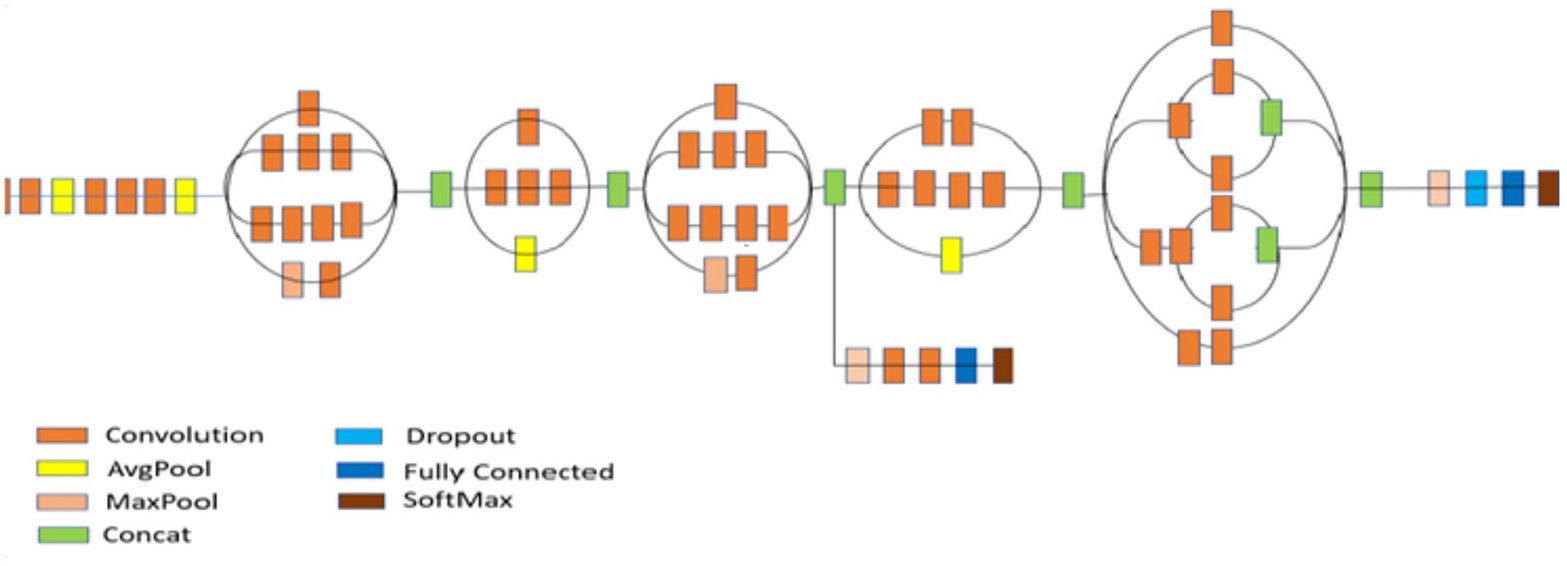
Architecture of inception V3 model (Szegedy et al., 2016).

The crucial phase in the evolution of CNN architectures is the development of inception networks. There are four variants, each with different levels of precision and performance. The creation of the Inception V3 model excelled in ImageNet, with a small error rate (Szegedy et al., 2016). In the second version, the supplementary classifiers included in the Inception architecture do not begin to participate until the training phase is complete. Minor modifications were made to the Inception V3. There were 42 layers in Inception V3. Normalization of auxiliary classifiers, label smoothing, 7 × 7 convolution, and the RMSProp optimizer are additional features of the earlier versions. Figure 9 depicts the structure of the Inception v3 model.

## 3. Results

In this work, we developed a fully automated categorization method for brain tumors. The raw MRI images were first preprocessed, the borders of the brain tissue were mechanically identified, and images were cropped. The dataset was subsequently increased using a data-augmentation technique. The processing load was reduced, and successful results were obtained with little data using the transfer learning method.

### 3.1. Performance metrics

A performance of the classifier was measured using a variety of parameters. The most frequently used metric was accuracy. The ratio of the number of correctly classified samples to the total number of data points was used to measure the classification accuracy, as represented in Eq 8.


(8) $$\begin{array}{c} Accuracy=\frac{Number\;of\;correctly\;classified\;samples}{Total\;number\;of\;samples} \end{array}$$

If the testing dataset comprises the same number of samples from all classes, classification accuracy is an appropriate metric for characterizing performance. Confusion matrices were used to assess the system working with an unbalanced dataset. True and false catalogs are presented as tables in a confusion matrix. The following outcomes are represented by values in a confusion matrix: when the original positive data are accurately classified as positive, it is referred to as a true positive (TP); when the initial positive data are incorrectly classified as a negative, it is referred to as a false negative (FN). When the negative data are properly categorized as negative, it is referred to as a true negative (TN), and false negatives are created when the first negative data are incorrectly labeled as positive (FP). Different metrics were created using the values obtained from the confusion matrix to represent the performance of the classifier.

These results were used to calculate recall (sensitivity), which is represented by Eq 9. The recall shows how well a classification system can identify true positives.


(9) $$\begin{array}{c} Recall=\frac{TP}{TP+FN} \end{array}$$

Equation 10 represents the precision. Precision is a measure of how well a classification can weigh false positives.


(10) $$\begin{array}{c} Precision=\frac{TP}{TP+FP} \end{array}$$

The harmonic mean of the above two metrics was utilized as the F1- score to represent the balance between precision and recall. Eq 11 was used to calculate the F1 score.


(11) $$\begin{array}{c} F1\;Score=\;2^{*}\frac{Precision^{*}Recall}{Precision+Recall} \end{array}$$

The precision, Recall, and F1 score values were assessed using the accuracy parameters for each model utilized in this investigation.

### 3.2. Findings

Four distinct pre-trained models were used to classify the dataset of 305 brain MR images. Each model used had the same batch size, number of epochs, and learning rate optimization as all other external factors. Thirty percent of the dataset was used for testing, whereas 70% was used for training. A validation set was created using 30% of the test set. For each model, epoch-based accuracy and loss graphs were obtained. In addition, the effectiveness of the individual models was evaluated in relation to the given metrics.

#### 3.2.1. VGG-19 model

**Figure 10:**
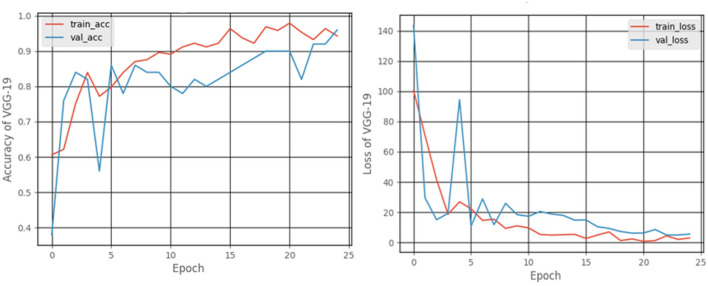
Accuracy and loss graphs for the VGG-19 model.

Figure 10 shows the accuracy and loss graphs created during the training and testing, procedures. At the end of 25 epochs, the accuracy was 99.48%, Recall was 98.76%, precision was ~100%, and F1 score was 99.17%.

#### 3.2.2. VGG-16 model

**Figure 11:**
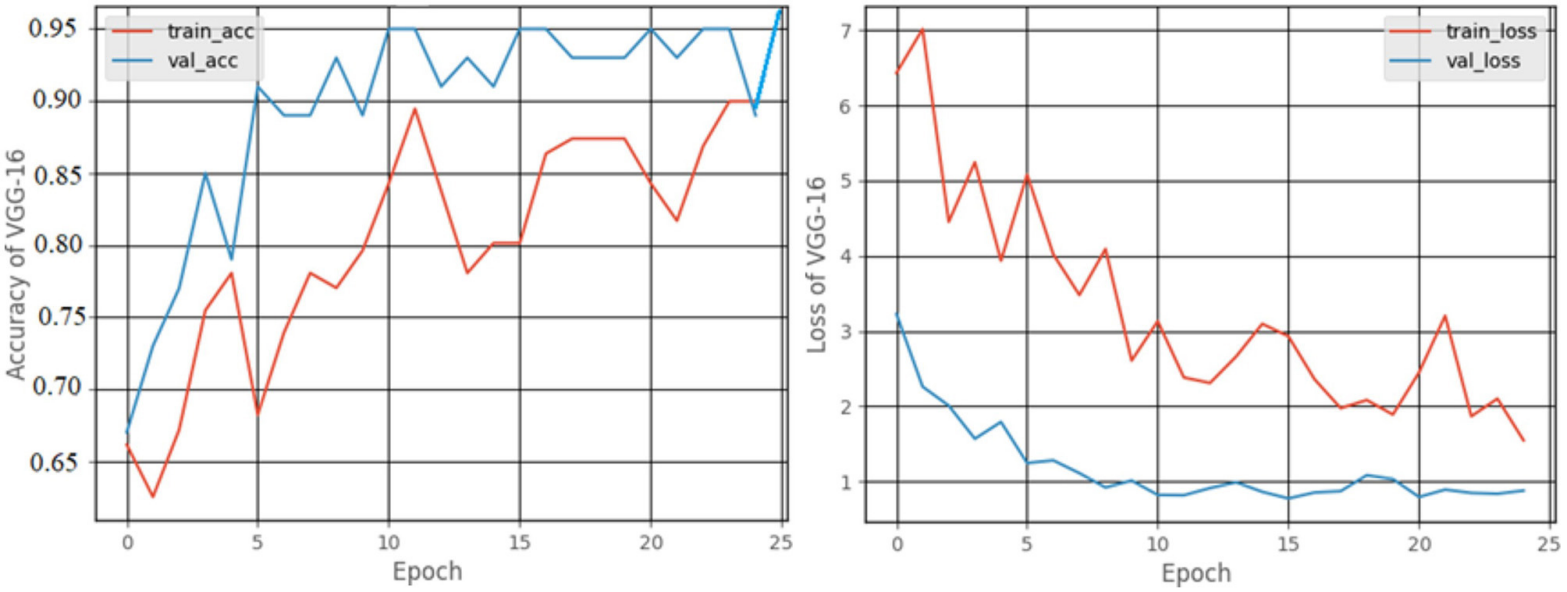
Accuracy and loss graphs for the VGG-16 model.

Figure 11 shows the accuracy and loss graphs created during the training and validation procedures. At the end of 25 epochs, the accuracy was 99%, recall was 98.18%, precision was approximately about 100%, and the F1 score was 99.08%.

#### 3.2.3. ResNet50 model

**Figure 12:**
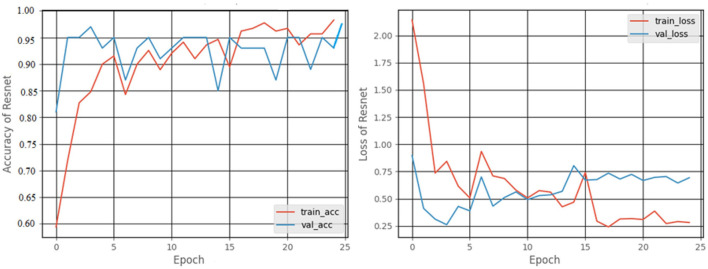
Accuracy and loss graphs of the ResNet50 model.

Figure 12 shows a graphical representation of the accuracy and loss created during the training and testing procedures. The accuracy attained at the completion of 25 epochs was 97.92%, recall was 87.7%, precision was 77.77%, and F1score was 82.24%.

#### 3.2.4. Inception v3 model

**Figure 13:**
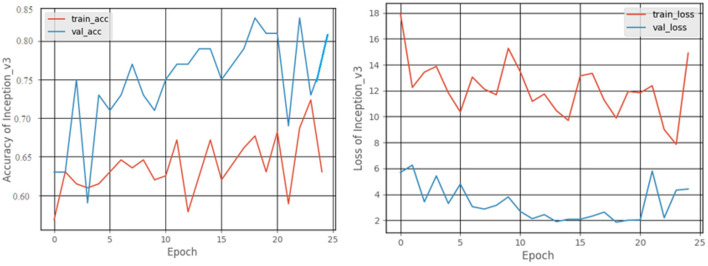
Accuracy and loss graphs of Inception V3 model.

The graphical records of the accuracy and loss obtained as an outcome of the training and validation processes are shown in Figure 13. At the end of 25 epochs, the Inception V3 model had an accuracy of 81.25% and, 63.25% for recall, a precision of 53.84%, and an F1 score of 58.16%.

**Figure 14:**
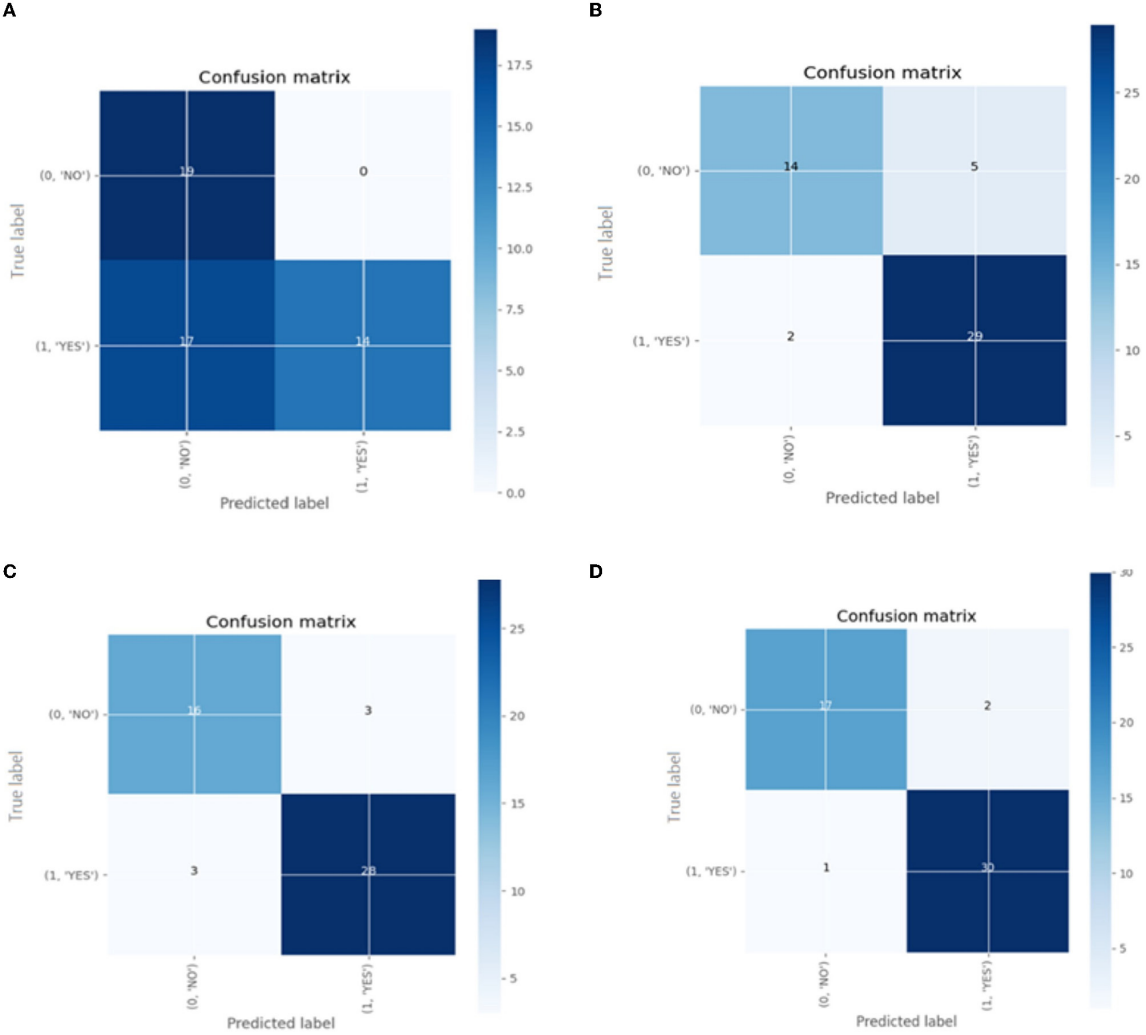
The confusion matrix for classification models **(A)** Inception V3 **(B)** ResNet50 **(C)** VGG-16 **(D)** VGG-19.

Confusion matrix for the different pre-trained models is shown in Figure 14.

**Figure 15:**
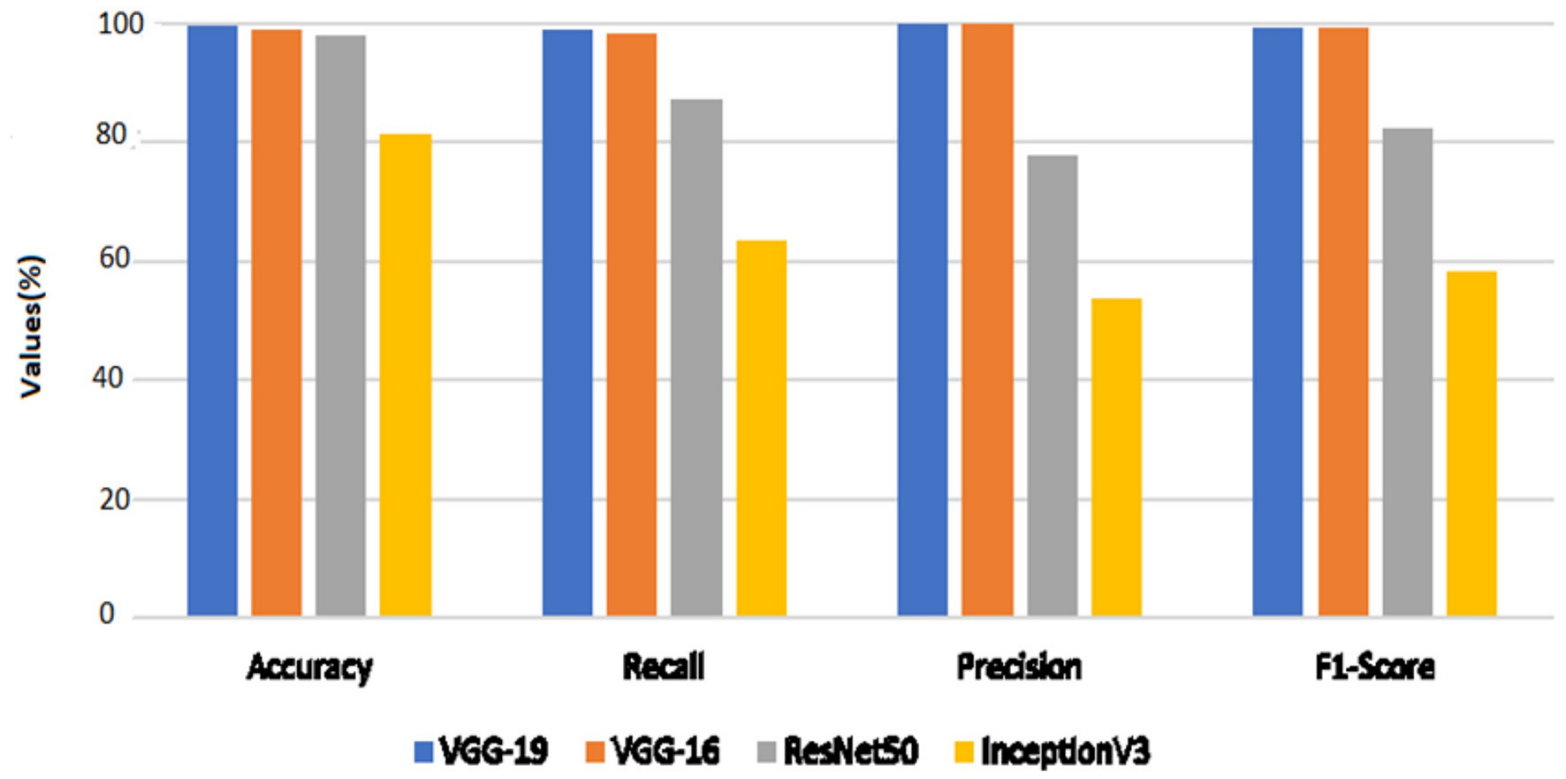
Evaluation metric comparison of various classification models.

The performance outcomes of the four pretrained models with an equal number of epochs are listed in Table 2. A comparison of the findings in Table 2 is presented in Figure 15.

**Table 2:** Performance metrics of classification models.

**Model**	**Accuracy (%)**	**Recall (%)**	**Precision (%)**	**F1-score (%)**
VGG-19	99.48	98.76	100	99.17
VGG-16	99	98.18	100	99.08
ResNet 50	97.92	87.27	77.77	82.24
Inception V3	81.25	63.25	53.84	58.16

### 4. Discussions

This section provides classification results on a dataset made publicly available by Chakrabarty on Kaggle.com and https://www.kaggle.com/datasets/navoneel/brain-mri-images-for-brain-tumor-detection. It also illustrates the comparison of the pretrained VGG-19, VGG-16, ResNet50, and Inception V3 DCNN models with other existing state-of-the-art artworks on pathological image classifications. A comparison was made in terms of the performance metrics reported in the literature, that is accuracy. The results are presented in Table 3.

**Table 3:** Comparison of the pre-trained models using transfer learning technique with existing state-of-the-art artworks.

**References**	**Methodology**	**Dataset**	**Accuracy (%)**
Zhang et al. (2013)	DWT + PCA + PSO optimized Kernel SVM	90 images	97.78
Yang et al. (2016)	DWT + BBO optimized SVM	90 images	97.78
Lu et al. (2017)	DWT + BAT optimized machine learning	132 images	98.33
Nayak et al. (2018)	Ripplet-II Features + PCA + ModifiedPSO based ELM	160 images	99.26
Zhang et al. (2018)	Pseudo Zernike moment + Kernel SVM	160 images	99.45
Khan et al. (2020)	KM clustering + Deep learning with data augmentation	220 images	94.06
Proposed work	VGG-19 with Transfer Learning	305 images	99.48
	VGG-16 with Transfer Learning		99
	ResNet50 with Transfer Learning		97.92
	Inception V3 with Transfer Learning		81.25

BBO, Biogeography based optimization; DWT, Discrete Wavelet Transform; ELM, Extreme Learning Machine; PCA, Principal Component Analysis; PSO, Particle swarm optimization; SVM, Support Vector Machine.

From Table 3, it can be concluded that the pretrained VGG-19 model with transfer learning attained an accuracy of 99.48% for brain tumor classification. It is superior to the techniques devised in works by Zhang et al. (2013, 2018), Yang et al. (2016), Lu et al. (2017) and Nayak et al. (2018), for which the accuracy values were 97.78%, 97.78%, 98.33%, 99.26%, and 99.45%, respectively. Moreover, the techniques devised in Zhang et al. (2013, 2018), Yang et al. (2016), Lu et al. (2017) and Nayak et al. (2018) used different feature extraction and selection techniques. The prime advantage of the pre-trained models with transfer learning in comparison to the existing works in Table 3 is that it needs no feature extraction mechanism and no intermediate feature selection phase.

When the models are represented side by side in terms of metric values, it can be understood that VGG- 19, which has an accuracy of 99.48%, performs the best on the dataset worked with.VGG-16, with 99% accuracy, is the second most effective pre-trained architecture. In comparison to these two models, the ResNet50 model displayed 97.92% accuracy, and Inception V3 displayed an accuracy of 81.25%. Regarding their capacity to identify true positives and eliminate false positives, the VGG- 19, and VGG-16 models exhibited better performance. By contrast, VGG-19 was condescending in terms of each metric.

**Figure 16:**
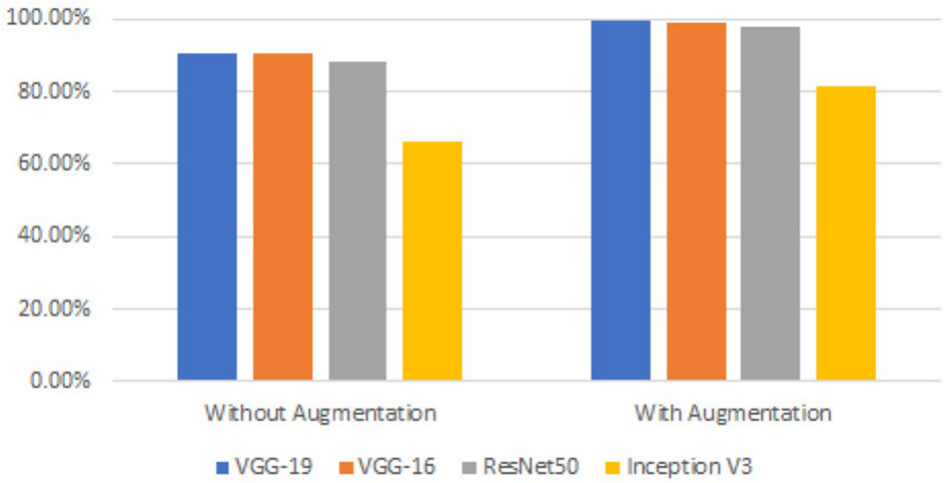
Comparison of model accuracies with and without augmentation.

We wanted to boost the success by utilizing the data augmentation technique because the dataset utilized in this work was unbalanced. Each model was run without applying data augmentation while maintaining the same other parameters to determine the impact of data increase on gaining the prediction. Table 4 shows a comparison of the accuracy values acquired with and without data augmentation. Figure 16 shows a comparison of observations from the results in Table 4.

**Table 4:** Accuracy values of classification models with and without data augmentation.

**Model**	**Without augmentation (%)**	**With augmentation (%)**
VGG-19	90.7	99.48
VGG-16	90.5	99
ResNet50	88.02	97.92
Inception V3	66.26	81.25

The results of the measurements revealed that the success of the model increased by up to 33.39% when employing the data augmentation technique. The majority of studies on the classification of MR brain images in the literature have used machine learning methods based on manually created characteristics. Manual feature extraction is labor-intensive and increases error rates. The capacity of deep learning for self-learning makes it possible for the features of MR images to be automatically discovered. In Table 5, the results of this study's classification of brain MR images using deep learning methods are compared with those of recent similar studies.

**Table 5:** Comparison of the study's findings with existing research.

**References**	**Dataset**	**Technique**	**Accuracy (%)**
Shahamat and Abadeh (2020)	140 MR images	Custom CNN	70
Zhang et al. (2020)	361 MR images	R-CNN	79
Naser and Deen (2020)	110 MR images	CNN-UNet	89
Zhou et al. (2019)	192 MR images	InceptionV3	69
Saxena et al. (2020)	3,064 MR images	VGG-16	90
Mlynarski et al. (2019)	285 MR images	CNN-UNet	80.92
Afshar et al. (2018)	3,064 MR images	CapsNet	90.89
Abiwinanda et al. (2019)	3,064 MR images	Custom CNN	84.19
Pashaei et al. (2018)	3,064 MR images	Custom CNN	81.09
Yang et al. (2018)	113 MR images	Google Net	86.7
Mostafiz et al. (2021)	274 MR images	Deep CNN	98.79
Ahmad and Choudhury (2022)	250 MR images	VGG-19	99.39
Proposed work	305 MR images	VGG-16	99
		VGG-19	99.48

The four pretrained models were implemented on the PYTHON 3.10.2 platform and executed on a system with an Intel(R) Core (TM) i5-1035G1 CPU, 8 GB RAM, and 3.3 GHz. The computation time (training + testing) was 155 minutes on a single CPU system.

Table 5 demonstrates that the experiments in the literature utilizing various CNN models on varied-sized datasets range in accuracy from 70 to 90%. The VGG-19 model demonstrated maximum success with 99.48% accuracy. This investigation reveals that Saxena et al.'s analysis utilizing the same model and the best result of 99% produced using the VGG-16 model were both inferior to each other. InceptionV3 was the least accurate model, with an accuracy rate of 81.25%. Results from other studies that classified brain MR images using the Inception V3 model ranged from 55 to 69% (Saxena et al., 2020).

## 5. Conclusions

The excellent image quality of MRI makes it easier to identify abnormalities in the brain. Radiologists typically evaluate MR images and diagnose tumors. However, it is now challenging to manually evaluate vast amounts of data collected in a reasonable amount of time, owing to advancements in medical imaging techniques. Deep learning has garnered considerable interest in the field of medical image analysis, owing to its ability to accurately discover complicated associations. CNNs are frequently used in deep-learning-based brain tumor segmentation. Transfer learning is used to enhance the efficiency of data processing. Remarkable success was attained when the workload of the new model was reduced by the transfer of the learned parameters. Additionally, transfer learning ensures success even when limited training data are available.

In this work, classifications were performed on a dataset of 305 brain MRI images with and without tumors using pre-trained VGG-19, VGG-16, ResNet50, and Inception V3 models. Raw MR images were preprocessed to ease the effort required during training. Raw images were cropped and scaled after the margins of brain tissue were established. Data augmentation was used to balance class distributions in the dataset. Accuracy, recall, precision, and F1-score measures were used to evaluate the classification process using four alternative models. With 99.48% accuracy, 98.76% recall, 100% precision, and 99.17% F1 score, the VGG-19 model demonstrated the greatest success. With 99% accuracy, 98.18% recall, 100% precision, and 99.08% F1 score, the VGG-16 model was next. The ResNet50 and Inception V3 models presented accuracy of 97.92 and 81.25%, respectively. In this study, as in related studies, the Inception V3 model had the lowest success rate. The results of this investigation were compared with those of recent related studies published in the literature. The most effective model in this analysis, VGG-19, was found to be more effective than those in previous studies in the literature. This work demonstrated that the transfer learning process produces good outcomes with little data and few epochs.

A limitation of the proposed work is that the training time is very large. The processing of a large amount of data is required to study MR images. Computers' graphics processing unit (GPU) offers better performance for this data-processing activity than a central processor unit (CPU). Hybrid DNNs and State-of-the-art (SOTA) DNN models such as Efficient Net, Mobile Net and DenseNet169 fed with YOLO can be used to improve the classification performance of the system.

## Data availability statement

The datasets presented in this study can be found in online repositories. The names of the repository/repositories and accession number(s) can be found in the article/supplementary material.

## Author contributions

Conceptualization, methodology, analysis, and paper writing by SK. Supervision, reviewing, and paper editing by YK. Both authors contributed to the article and approved the submitted version.
